# Multiple early gastric cancer with duodenal invasion

**DOI:** 10.1186/1477-7819-5-125

**Published:** 2007-10-30

**Authors:** Akihisa Matsuda, Shunji Kato, Masaichi Furuya, Yasuhito Shimizu, Tetsuya Okino, Junpei Sasaki, Takashi Tajiri

**Affiliations:** 1 Department of Surgery, Chikusei City Hospital, 1658 Tamado, Chikusei-shi, Ibaraki 308-0847, Japan; 2 Surgery for Organ Function and Biological Regulation (Department of Surgery), Graduate School of Medicine, Nippon Medical School, 1-1-5 Sendagi, Bunkyo-ku, Tokyo 113-8603, Japan

## Abstract

**Background:**

Early gastric cancers with duodenal invasion are rare, and no previous case of multiple early gastric cancer, one invading the duodenal bulb, has been reported.

**Case presentation:**

A 79-year-old woman was investigated for upper abdominal discomfort. Endoscopic examination revealed an irregular nodulated lesion in the antrum area, and a reddish aggregated-type semi-circumferential nodulated lesion extending from the prepyloric area to the duodenal bulb through the normal mucosa with the antrum lesion. Biopsy revealed a tubular adenoma for the antrum lesion and a well-differentiated tubular adenocarcinoma for the prepyloric lesion. Distal gastrectomy with sufficient duodenal resection was performed. Microscopically, the antrum lesion appeared as a papillary adenocarcinoma, and the prepyloric lesion as a mainly papillary adenocarcinoma which partially invaded the submucosa without any sequential elongation for endoscopic findings. The lesion extended into the duodenal bulb, and was 12 mm in length from the oral end of Brunner's gland's area and limited within the duodenal mucosa.

**Conclusion:**

Here, we present an unusual case of multiple early gastric cancer, one of which invaded the duodenum with relative wide mucosal spreading. This case illustrates that even early stage cancers located in the gastric antrum, particularly in the prepyloric area can invade the duodenum directly.

## Background

Advanced gastric cancer of the antrum occasionally invades the duodenum beyond the pyloric ring. These cases are reported to have a poor prognosis [1,2]. However, early gastric cancers with duodenal invasion are rare, with an incidence ranging from 0.5% to 1.8% of all gastric cancer cases [3-5]. In this report, we describe a patient with two early gastric cancers, one of which invaded the duodenal bulb, and discuss the clinicopathological features of early gastric cancer cases with duodenal invasion.

## Case presentation

**Figure 1:**
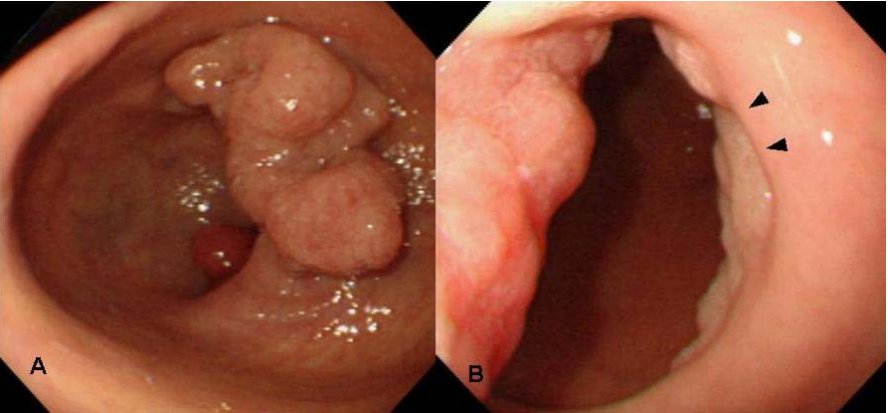
**a, b**. Endoscopic examination showed an irregular nodulated lesion in the antrum area (**a**) and a reddish aggregated-type semi-circumferential nodulated lesion extending from the prepyloric area to duodenal bulb through the normal mucosa with the former lesion (*arrowheads *show the p-ring) (**b**).

**Figure 2:**
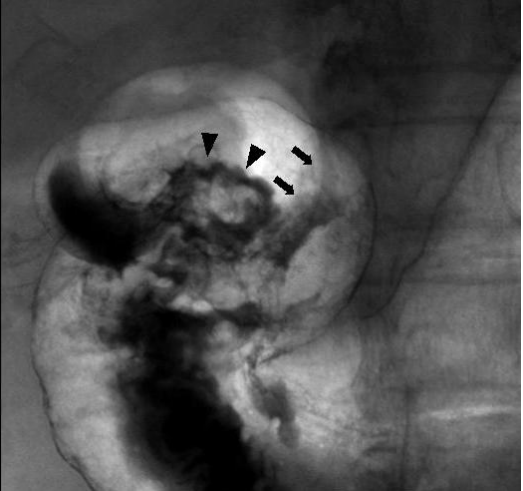
The double-contrast picture showed an irregular nodulated lesion from the prepyloric area to the duodenal bulb (*arrowheads *show extension to the duodenal bulb. *arrows *show the p-ring)

**Figure 3:**
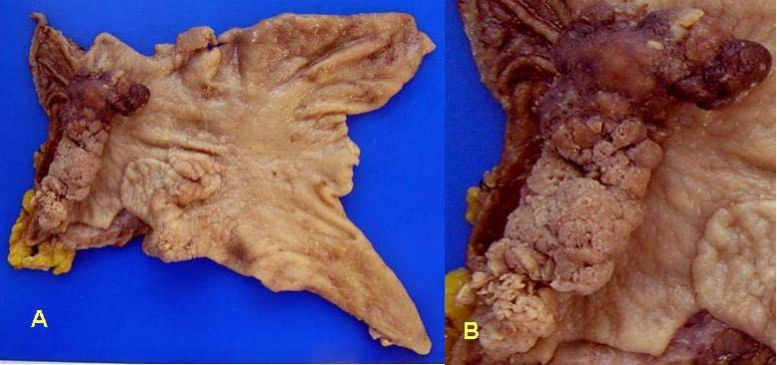
**a, b**. Microscopically, the resected specimen showed two independent irregular nodulated lesions. The oral-side lesion extending superficially, is located in the antrum area and is 70 × 40 mm (**a**). The anal-side semi-circumferential lesion is 70 × 25 mm and located mainly in the prepyloric area and duodenal bulb beyond the pyloric ring (**b**).

A 79-year-old Japanese woman complained of upper abdominal discomfort during postoperative follow-up for breast cancer in March 2005. She had no remarkable past history other than breast cancer and her relatives have not been suffered no malignant diseases. On physical findings, her temperature was 36.6°C, and there was no palpable abdominal mass. No sign of lymphadenopathy was found. Initial laboratory testing revealed no abnormalities other than a high serum concentration of CA19-9 (42.4 U/ml; normal, <37 U/ml) (Table 1). Endoscopic examination revealed an irregular nodulated lesion in the antrum area (Figure 1a), and a reddish aggregated-type semi-circumferential nodulated lesion extending from the prepyloric area to the duodenal bulb through the normal mucosa with the antrum lesion (Figure 1b). The biopsy specimens revealed a tubular adenoma from the antrum lesion and a well-differentiated tubular adenocarcinoma from the prepyloric lesion. A double-contrast picture showed an irregular nodulated lesion extending from the prepyloric area to the duodenal bulb; however, no hardening of the gastric wall was detected on barium study (Figure 2). Metastatic workup was negative. Distal gastrectomy with sufficient distal surgical margin, 30 mm from pyloric ring, and lymph node dissection (D2) was performed in April 2005. We adopted Roux-en Y reconstruction which is capable of cancer free anastomosis even if duodenal resection reached broad. The intraoperative distal surgical margin was negative. Macroscopically, the resected specimen showed two independent polypoid lesions, an oral-side lesion of 70 × 40 mm located in the antrum area extending superficially and an anal-side semi-circumferential lesion of 70 × 25 mm located mainly in the prepyloric area and duodenal bulb beyond the pyloric ring. The intervening mucosa between the two lesions was normal (Figure 3ab).

**Table 1:** Laboratory data on admission

**Laboratory parameter**	**Value**	**Parameter**	**Value**
White blood count	42 × 10^2^/μL	Total bilirubin	1.1 mg/dL
Red cell count	415 × 10^4^/μL	Sodium	142 mEq/L
Hemoglobin	13.2 gm/dL	Potassium	4.1 mEq/L
Hematocrit	39.6%	Chloride	103 mEq/L
Platelets	22.5 × 10^4^/μL	Blood urea nitrogen	17.5 mg/dL
SGOT	36 IU/L	Creatinine	0.7 mg/dL
SGPT	34 IU/L	Total protein	7.7 gm/dL
LDH	206 IU/L	Albumin	4.7 gm/dL
Amylase	111 IU/L	C reactive protein	0.03 mg/dL
CPK	216 IU/L	CEA	1.2 ng/ml
CA 19-9	42.4 U/L		

**Figure 4:**
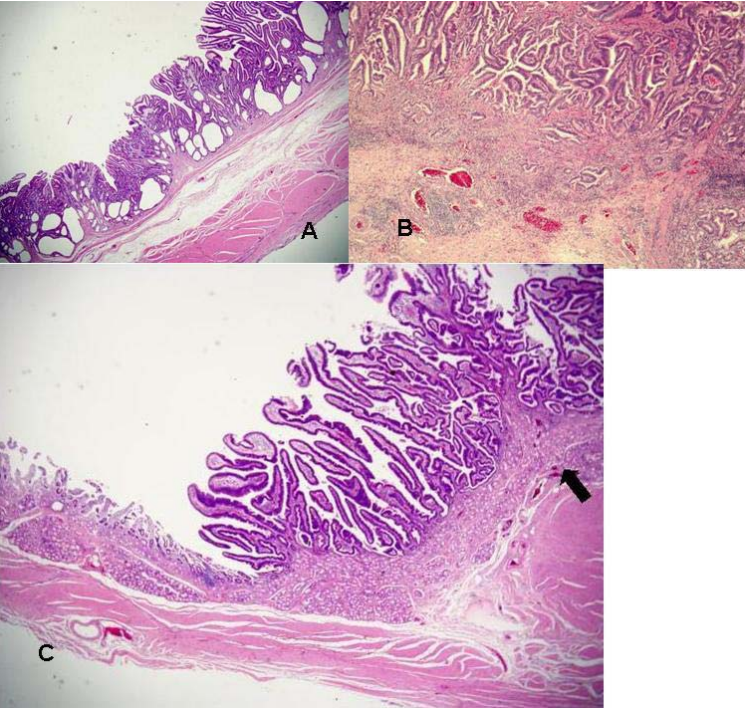
**a-c**. Microscopically, the antrum lesion showed a well-differentiated papillary adenocarcinoma limited within the gastric mucosa (HE stain. × 10) (**a**). The prepyloric lesion showed a mainly well-differentiated papillary adenocarcinoma partially invading the submucosa (HE stain. × 20) (**b**). The lesion extending into the duodenal bulb was limited within the duodenal mucosa and showed an identical histology to the prepyloric part (HE stain. × 10). The *arrow *shows the beginning of Brunner's glands (**c**).

**Figure 5:**
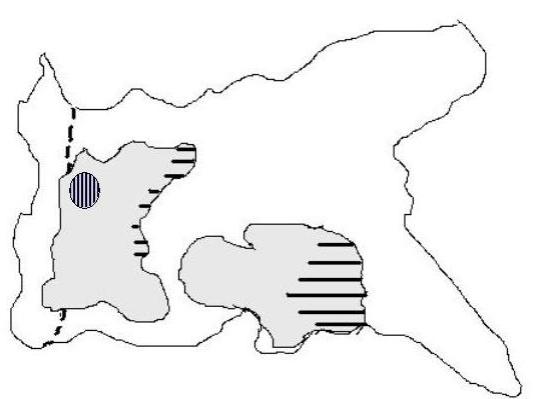
Schematic representation of the resected specimen. Horizontal lines show adenoma components of each tumor. Vertical lines show submucosal invasion of the prepyloric lesion, while dotted lines show the pyloric ring.

The resected specimen was examined histologically to determine the depth and mode of invasion, metastasis to lymph nodes, histologic type, and invasion to the duodenum according to the general rules established by the Japanese Gastric Cancer Association [6]. Microscopically, the antrum lesion showed a papillary adenocarcinoma with high-grade atypia limited within the gastric mucosa without vessel invasion (Figure 4a). The prepyloric lesion showed a mainly papillary adenocarcinoma mixed with tubular and villous formations which partially invaded the submucosa without vessel invasion (Figure 4b). The cancerous lesion extended into the duodenal bulb, and was 12 mm in length from the oral end of Brunner's gland's area, and limited within the duodenal mucosa. This lesion showed an identical histology to the prepyloric part (Figure 4c). The two lesions were classified as intestinal type and separated by at least 20 mm of intervening normal mucosa. The back ground mucosa of these lesions was mild atrophic gastritis and intestinal metaplasia. Helicobacter pylori infection was detected by histological findings. The distal margin of the surgical specimen, 4 mm in length, was negative. A schematic representation of the resected specimen is shown in Figure 5. No dissected lymph nodes showed metastasis. We finally diagnosed the lesions as two early gastric cancers, as follows. Antrum lesion: L, pap, type 0 I, pT1(M), ly0, v0, pPM(-), pDM(-), pN0, sH0, sP0, sM0, CY0; and prepyloric lesion: LD, pap, type 0 I, pT1(SM), ly0, v0, pPM(-), pDM(-), pN0, sH0, sP0, sM0, CY0, stage IA. The postoperative course was uneventful and the patient was discharged 38 days after admission. No recurrence or metastasis has been detected during three years of follow-up after the operation.

## Discussion

Here, we present an unusual case of multiple early gastric cancer, one of which invaded the duodenum with relative wide mucosal spreading. Although advanced gastric cancer with duodenal invasion is widely reported, cases of early gastric cancer with duodenal invasion are rare, with an incidence ranging from 0.5% to 1.8% of all gastric cancer cases [3-5]. 34 cases of early gastric cancer with duodenal invasion have been reported previously, to our knowledge, no case of multiple early gastric cancer has been reported (Table 2). Multiple gastric cancer is defined generally by Moertel's criteria [7]. Moertel reported the incidence of multiple gastric cancer is 2.1%. Recently, the incidence is increasing up to 6% approximately with an improvement of diagnostic technique [8].

**Table 2:** Reported cases early gastric cancer with duodenal invasion

Authors	Year	Size (mm)	Location	Length of invasion (mm)	Histologic type (duodenum)	Type	Depth (duodenum)	N
Ishii	1975		LD, Circ	7	Tub	Depressed	m(m)	0
		30 × 15	LD	5	Pap	Elevated	sm(m)	0
Kuwayama	1976	40 × 35	LD, ant-less		Pap (por)	Depressed	m(m)	0
Uchida	1979	32 × 25	LD, Circ	7	Por	Depressed	m(m)	0
		35 × 21	LD	2	Tub1	Mixed	sm(sm)	0
		30 × 15	LD	5	Pap	Elevated	sm(m)	0
Kuwata	1981		LD, less	5	Sig	Depressed	sm(m)	0
			LD, gre	6	Tub1 (pap)	Elevated	sm(m)	0
			LD, less	2	Pap	Elevated	sm(m)	0
			LD post	1	Tub2	Mixed	sm(m)	0
		30 × 15	LD, less	2	Tub2	Mixed	sm(m)	0
		35 × 21	LD, less	1	Tub2	Depressed	sm(m)	0
		30 × 15	LD, less	5	Pap	Mixed	sm(m)	0
Katoh	1993	68 × 36	LD, circ	16	Tub1(pap)	Elevated	m(m)	0
Nakazawa	1994	10 × 10	LD, ant-gre	3	Tub1	Depressed	m(sm)	0
Boku	1996	70	LD, circ	40	Tub1	Elevated	sm(m)	0
Itoh	1996	45 × 35	LD, less	25	Tub1(pap)	Mixed	sm(m)	0
Matsumoto	2000	25 × 9	LD, less	3	Sig	Elevated	m	
		25 × 10	LD, gre-post	10	Sig	Elevated	sm	
		30 × 13		3	Mod	Elevated	m	
		65 × 23		3	Mod	Elevated	m	
		45 × 45		5	Mod	Superficial	sm	
Yasuda	2000	75 × 15	LD, circ	11	Sig(por)	Depressed	m(m)	0
Nakayama	2001	85 × 75	LD circ	38	Tub1	Mixed	sm(m)	0
Koufuji	2003	70 × 50	LD circ	2	Tub1	Mixed	sm	0
		90 × 55	LD, circ	2	Tub1	Elevated	sm	0
		120 × 98	LMD, circ	2	Sig	Depressed	m	0
		103 × 102	LMD, circ	8	Sig	Depressed	m	0
		55 × 24	LD, less	3	Sig	Depressed	sm	1
		30 × 20	LD gre	2	Tub1	Depressed	sm	1
		52 × 30	LD, less	5	Tub2	Depressed	sm	0
		57 × 33	LD circ	3	Sig	Depressed	m	0
		40 × 38	LD gre	5	Por2	Depressed	sm	1
		80 × 65	LD, circ	2	Sig	Depressed	sm	2
Our case	2007	30 × 15	LD, circ	12	Pap	Elevated	sm(m)	0

Clinically, definition of the border between the stomach and the duodenum is contentious. Although the pyloric ring is composed of gastric tissue macroscopically, Brunner's glands are present beginning in the mucosa over the pyloric ring. Clinicopathological studies of duodenal invasion suggest the duodenum starts at the point where Brunner's glands first occur [4,9]. The pyloric ring is the contact point between two different types of mucosa; hence, Brunner's glands seem to act as a protective barrier against cancer invasion [4]. Another report showed that the presence of extremely dense connective fibers in the subepithelial proper mucosal layer can prevent the direct extension of mucosal carcinoma into the duodenum [10]. These theories are thought to explain the rarity of early gastric cancer with duodenal invasion, at least in part [11]. However, the increasing number of such cases attests to the ability of it gastric cancer cells can to directly invade the duodenum in the mucosal layer [4,12]. Our case also showed direct mucosal invasion into the duodenum.

Preoperative diagnosis of early gastric cancer with duodenal invasion is difficult. Nakayama *et al*. [13] reviewed 24 cases in Japan and found that duodenal invasion was preoperatively detectable in only four, all of which were well-differentiated and of the elevated type. The microscopic extension of cancer cells in poorly-differentiated cases is often infiltrative and broader than the macroscopic extension, hampering the detection of duodenal invasion of poorly differentiated early gastric cancer, particularly for small lesions.

Regarding the length of duodenal invasion, most early gastric cancer cases reported to date have shown invasion of less than 10 mm [3,13]. This is another reason for the difficulty of detecting duodenal invasion preoperatively. In our case, however, invasion extended 12 mm into the duodenum from the beginning point of Brunner's glands. Uchida *et al*. [14] reported that papillary-type gastric cancer cells have a high affinity for the duodenal mucosa and that papillary-type gastric adenocarcinoma occasionally shows transient formation in the duodenal mucosa. These findings may help explain the broad duodenal invasion of over 10 mm in our case. Besides, most cases of early gastric cancer with duodenal invasion is limited to the mucosal layer of the duodenum, only 2 cases with submucosal invasion into the duodenum have been reported previously (Table 2). The cases of submucosal invasion into the duodenum do not always show the longer invasion into duodenum compared with mucosal invasion cases. This finding indicates that, as in the reported advanced cases with duodenal invasion, the depth of duodenal invasion does not independently affect the length of duodenal invasion [14]. Therefore, these imply us that, it is difficult to anticipate the extent of duodenal invasion, pre and intra operative close observation for duodenal invasion is indispensable to keep the distal surgical margin negative with sufficient duodenal resection.

Koufuji *et al*. [3] reported four cases of early gastric cancer with duodenal invasion which showed lymph node metastasis. All the four cases showed just one regional lymph node metastasis. Three of four cases metastasize to No. 6 and one case to No. 12 lymph node. Nevertheless, the small number of reported cases has prevented any clear understanding of whether early gastric cancer with duodenal invasion has a higher incidence of lymph node metastasis than that without duodenal invasion. Although the reported incidence of lymph node metastasis from early gastric cancer with submucosal invasion is approximately 20%, our present case showed no metastasis [15].

## Conclusion

A greater number of cases should help clarify the clinicopathological findings of early gastric cancer with duodenal invasion. The present case emphasizes that even early stage cancers located in the gastric antrum, particularly in the prepyloric area can invade the duodenum directly.

## Competing interests

The author(s) declare that they have no competing interests.

## Authors' contributions

AM, YS, TO, JS and MF carried out the surgical procedures. SK and TT revised and finally approved the manuscript for been published. All authors read approved the final version of the manuscript.
